# Credibility of Pont’s index in Egyptian population

**DOI:** 10.1186/s12903-024-04715-7

**Published:** 2024-08-29

**Authors:** Esraa Khaled Zahw, Nehal Fouad Albelasy, Ahmed Maher Fouda

**Affiliations:** 1 Clinical Demonstrator, Department of Orthodontics, Faculty of Dentistry, Mansoura University, Mansoura, Egypt; 2 Assistant Professor, Department of Orthodontics, Faculty of Dentistry, Mansoura University, Mansoura, Egypt; 3 Associate Professor, Department of Orthodontics, Faculty of Dentistry, Mansoura University, Mansoura, Egypt

**Keywords:** Pont’s index, Transverse deficiency of the maxilla, Arch width, Model analysis

## Abstract

**Background:**

Many indices have been suggested to help orthodontists in predicting the ideal dental arch width. One of these was Pont’s index which was established by Pont. He suggested equations to predict the ideal maxillary dental arch width (interpremolar and intermolar) from the combined mesiodistal width of the maxillary incisors. This study aimed to test the applicability of Pont’s index as an orthodontic diagnostic tool in Egyptian population and to compare the results with those obtained from studies of different ethnic subjects.

**Methods:**

This study was performed using dental casts of 184 Egyptian individuals (82 males and 102 females; age range, 19–24 years). The casts were divided into 46 casts with normal occlusion, 46 casts with class I, 46 casts with class II and 46 casts with class III malocclusion, according to Angle’s classification. Alginate impressions were taken for all patients and poured immediately using dental plaster. The real models were transformed into digital models using three-dimensional laser scanner to allow digital model analysis. Predicted arch widths were calculated using Pont’s equations. The predicted values were compared to the measured values.

**Results:**

Intra class correlation coefficient (ICC) (absolute agreement) between measured and predicted arch widths was determined. Poor absolute agreement was found between measured arch width values and the corresponding values calculated according to Pont’s index.

**Conclusion:**

According to the results of this study, Pont’s index is not a reliable method for predicting the ideal dental arch widths in Egyptian populations.

## Introduction

One of the most common oral health issues is Malocclusion (MO) which is prevalent in most countries [1, 2]. MO, which affects the maxilla or mandible, can occur in three planes of space: sagittal, vertical, and transverse. The most common of them are transverse alterations [3]. About 8–18% of people have transverse plane discrepancy [4]. The decrease in the transverse dimension of the maxillary arch is known as a transverse deficiency of the maxilla (TDM) [5]. Several indices have been proposed as a guide to help in diagnosis of TDM. One of the most popular indices of TDM was provided by Pont [6].

In 1909, Pont [6] suggested a method that he believed it could be useful in determining the ideal dental arch width and the amount of maxillary expansion necessary to relief dental crowding. He performed his work on a group of French population. He believed that there was a strong relationship between the combined mesiodistal (MD) widths of the maxillary four incisors and the dental arch width in premolar and molar areas in ideal, uncrowded dentition [7]. He divided the average sum of incisors (SI) by the average inter-premolar width (IPW) and inter-molar width (IMW) to obtain the premolar and molar index numbers, respectively. The sample size and inclusion criteria weren’t mentioned.

The formula was then adapted to arch width prediction by transposition: the predicted IPW = SI x 100/80 and the predicted IMW = SI x 100/64. Therefore, the practitioner only needs to know SI to calculate the arch widths. If the observed value is smaller in comparison with the predicted value, expansion is required to allow widening of the arch form to this calculated width so that the dentition could all line up precisely [8]. Pont [6] came to the conclusion that his study should be carried out on several racial and ethnic backgrounds in order to verify and correct. Numerous researchers have examined this index reliability and validity across a range of demographics. These researchers’ findings can be categorized into two opposing groups. Some researchers [9–14] disagreed with it while others [15, 16] supported its use. No previous study was carried out to evaluate Pont’s index in the Egyptian individuals so the goal of this study was to assess the validity and reliability of Pont’s index in calculating the dental arch width in normal occlusion (NO) and different classes of dental MO in the Egyptian population.

## Materials and methods

### Sample size calculation

Sample size was calculated by using Power Analysis and Sample Size (PASS) Software (version 15, 2017). NCSS, LLC. Kaysville, Utah, USA.

### Pearson’s correlation

A sample size of 184 subjects achieves 93% power to detect a difference of -0.25 between the null hypothesis correlation of 0.00000 and the alternative hypothesis correlation of 0.25 using a two-sided hypothesis test with a significance level of 0.05. The null hypothesis was that there was no difference between measured and calculated IPW & IMW based on Pont’s equations, while the alternative hypothesis based on multiple previous studies on different populations including Celebi et al. [11] in Turkey, Al-Omari et al. [9] in Jordan, Alam et al. [17] in Bangladesh, and Rathi et al. [18] in Pakistan indicated low-strength association (*r* = 0.25) and poor absolute agreement (ICC = 0.25) between estimated and measured arch widths.

### Participant selection

This study was performed using dental casts of 184 Egyptian individuals (82 males and 102 females; age range, 19–24 years to ensure end of transverse growth in males according to Cortella et al. [19]). Dental casts were randomly selected from Mansoura University students and orthodontic patients in the Orthodontic Department Faculty of Dentistry, Mansoura University, Egypt. The dental ethical review committee of Faculty of Dentistry, Mansoura University, Mansoura, Egypt, gave its approval to this study (Code No. A04041022). All patients signed the informed consent to participate in this study. This study included four groups where the records were collected according to the inclusion criteria and divided into 46 casts with NO, 46 casts with class I MO, 46 casts with class II MO and 46 casts with class III MO to represent the three classes of malocclusion according to Angle’s classification (Class I, II and III).

### Participants and eligibility criteria: [9, 17]

The eligibility of the participants was determined by Ez^a^ and revised and confirmed by NA^b^ and AF^c^. Individuals of confirmed Egyptian ethnic background were included in the study who fulfilled the selection criteria; complete set of permanent dentitions from the right first molar on one side to the left first molar on the opposite side in both arches; no supernumerary teeth; no previous orthodontic treatment; no posterior crossbite; Minimal rotation; teeth were nearly aligned in the arch with no more than 1 mm crowding, bilateral class II canine and molar relationship in class II MO group and bilateral class III canine and molar relationship in class III MO group.

### The exclusion criteria

Individuals with extensive restorations that affected MD dimensions of teeth, attrition, erosion, abrasion, fractured crowns or congenitally deformed teeth, as peg shaped lateral incisors were excluded. Any medical or dental condition or oral habit which could affect jaw dimensions were excluded.

## Method

High quality Alginate impression material was used in the study to take impressions for all patients who fulfilled the selection criteria and the bite was recorded using dental wax to ensure accurate articulation of the study models. Study models were obtained by immediate pouring of impressions using dental plaster with good dimensional stability. The real study models were transformed into digital models with the use of a three-dimensional (3D) model laser scanner (Medit T310 | 3D scanners | Seoul, Republic of Korea). After finishing the scanning process, the selected STL file was imported for analysis process using 3shape Ortho Analyzer software, Copenhagen, Denmark.

### Steps of digital cast analysis

**Fig. 1:**
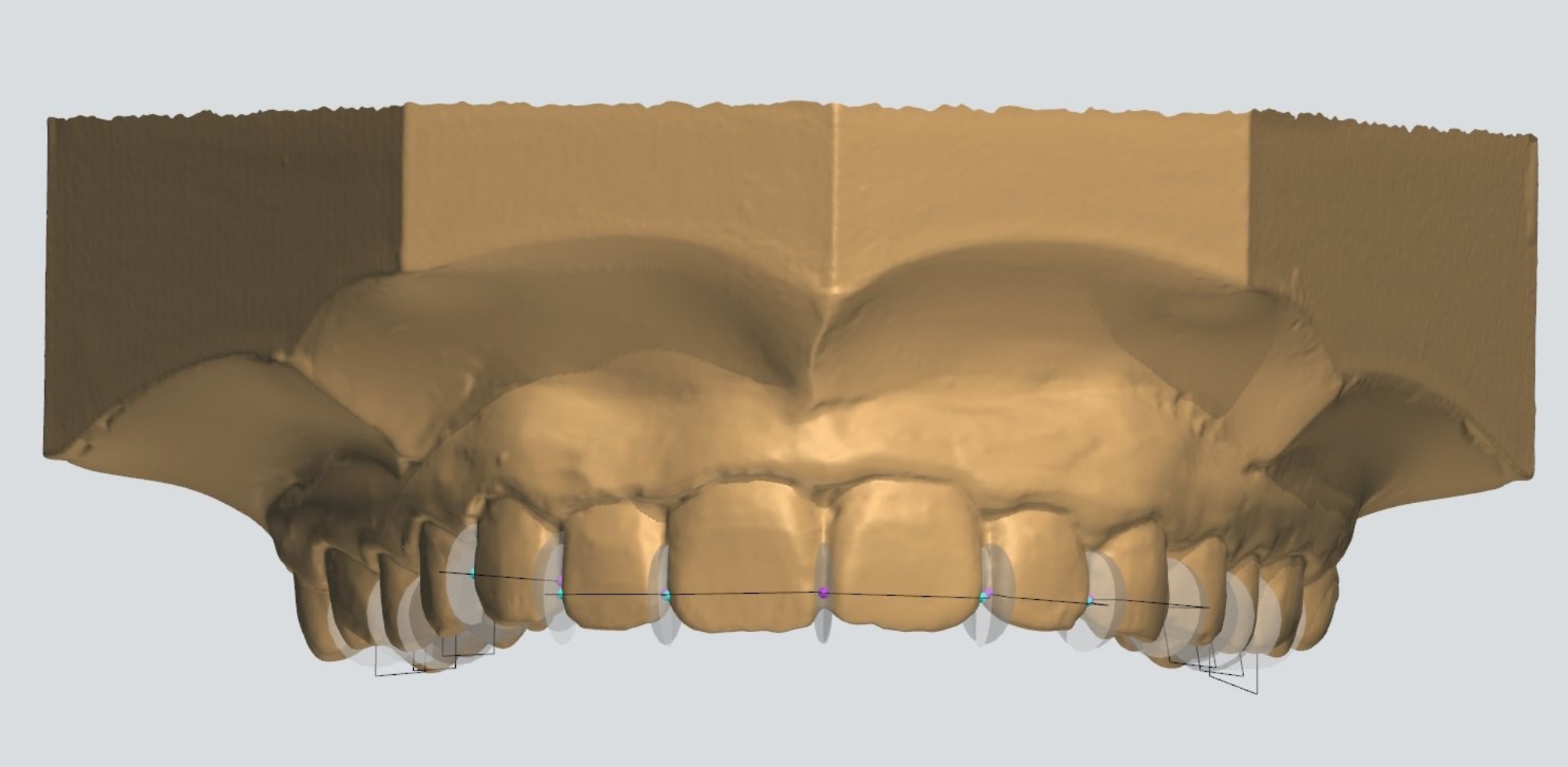
Frontal view of the MD width of the four maxillary incisors

**Fig. 2:**
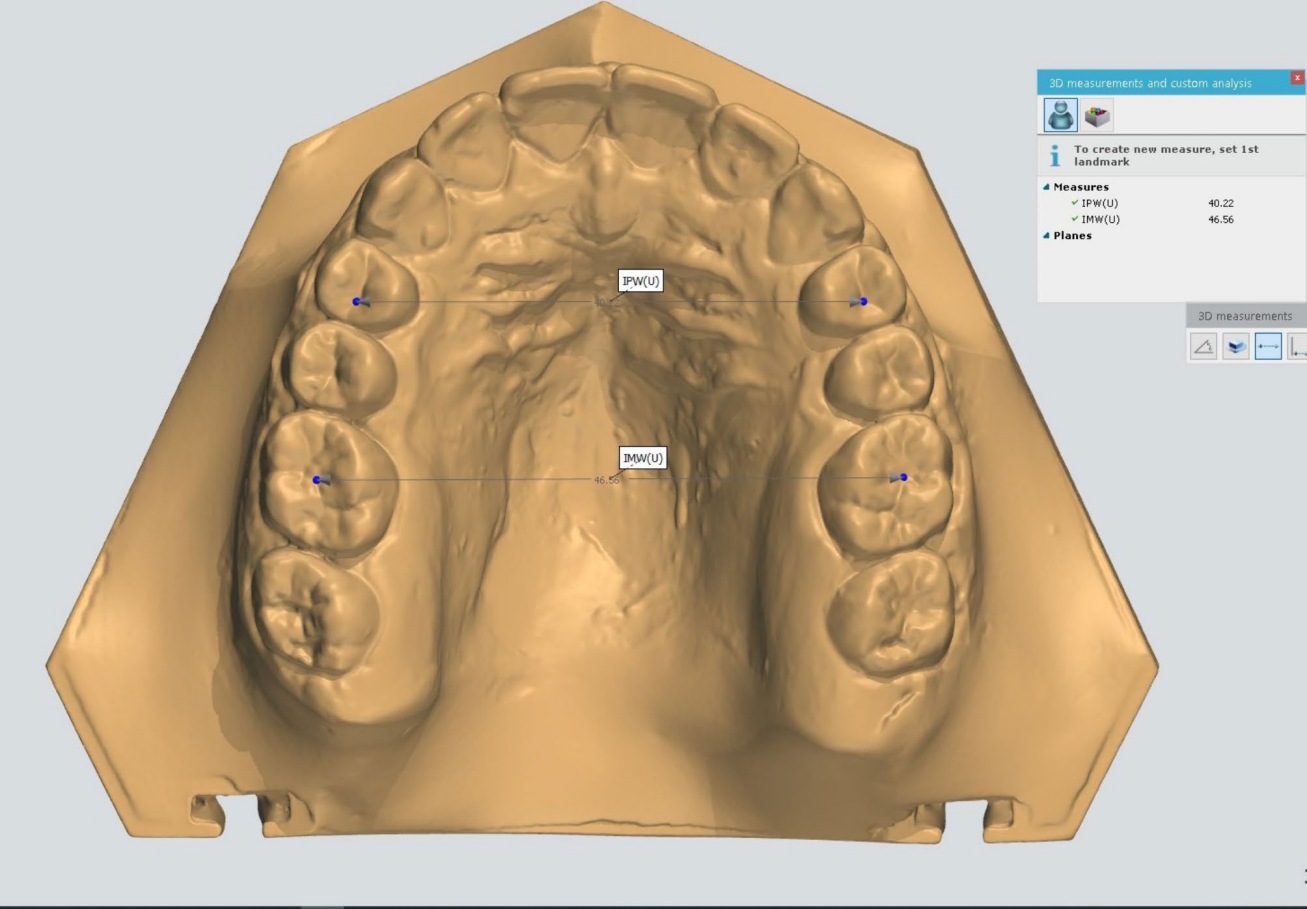
Occlusal view of the upper cast where the IPW was determined between distal pits of first premolars and IMW was determined between central fossae of first permanent molars

For each cast, the following steps were done by a well trained and experienced examiner (EZ^a^):

The MD widths of the maxillary incisors were determined. Measurements were recorded from the maximum MD dimension of every tooth from the frontal view (Fig. 1). From the occlusal view of the upper cast, the upper inter-premolar width (IPW) was measured between distal pits of first premolars and the upper inter-molar width (IMW) was measured between central fossae of first permanent molars (Fig. 2).

From the previous data, the sum of the MD dimension of the upper incisors (SI) was calculated for each cast and Pont’s equations were applied to calculate IPW & IMW as mentioned before.

### Error of the method

To ensure measurements accuracy and reliability, 4 weeks later, the measurements were retaken again for each cast by the same examiner (EZ^a^). The collected data was revised by NA^b^ and AF^c^.

### Statistical analysis

Data were entered and analyzed using IBM-SPSS software (version 27, 2020) & MedCalc Software (version 20, 2021). Quantitative data were initially tested for normality using Shapiro-Wilk’s test and the presence of significant outliers was tested for by inspecting boxplots. Intraclass correlation coefficient (ICC) was used as a reliability index in testing intra-rater reliability analysis (absolute agreement). The one-way ANOVA test was used to compare SI, measured and predicted IPW & IMW according to Pont’s index between males and females and between NO and different classes of dental MO in Egyptian individuals, followed by post-hoc Games-Howell test. ICC was used as a reliability index for measured-predicted reliability analysis (absolute agreement). The Bland-Altman plot was used to compare measured and predicted IPW & IMW. To ascertain whether there is a linear relationship between SI and measured IPW & IMW, the Pearson’s correlation test was employed. If *P*-value for any of the tests was ≤ 0.05, the outcomes were proved to be statistically significant. Simple linear regression test was used to predict IPW & IMW from SI. Regression equations were calculated.

## Results

### Testing reliability

Intraclass correlation coefficient (ICC) was used for intra-observer absolute agreement on cast measurements. For every parameter analyzed, there was a sufficient agreement between the initial and repeated measurements. There was no statistically significant difference between the two sets of measurements (*P*-value < 0.001). This indicates excellent intra-observer absolute agreement for all studied measurements (Table 1).

**Table 1:** Intra-observer reliability (absolute agreement) for all measured values

Source	ICC	95% CI for ICC		*p*-value
		Lower	Upper	
SI	0.988	0.972	0.994	**< 0.001**
Measured IPW	0.996	0.992	0.998	**< 0.001**
Measured IMW	0.998	0.996	0.999	**< 0.001**

Notes: ICC = intraclass correlation coefficient. CI = confidence interval. SI = sum of incisors. IPW = inter-premolar width. IMW = inter-molar width

Descriptive statistics (Mean and SD) and one-way ANOVA test was applied to compare normally distributed quantitative data between genders (Table 2) which revealed no significant difference between genders in SI, measured and predicted IPW & IMW (*P* < 0.05). The results showed that the average predicted IPW & IMW based on Pont’s equations are higher in comparison with the actual measured means for overall Egyptian sample, suggesting that Pont’s index overestimates IPW & IMW in Egyptians (Table 3). Comparison between the four groups; NO, class I, class II and class III MO using one-way ANOVA test revealed no significant difference between the four groups in SI and predicted IPW & IMW (Table 3). On the contrary, a statistically significant difference in measured IPW & measured IMW between the four groups was recorded (*P*-value ≤ 0.05). The measured IPW was lower in class II < class I < class III < NO and the measured IMW was lower in class II < class I and NO < class III (Table 3). Accordingly, post-hoc tests for significant results of one-way ANOVA showed a significant difference in class II vs. all other three groups for measured IPW. A statistically significant difference in class II vs. class III for measured IMW (Table 4).

**Table 2:** Mean, SD and *P*-value of one-way ANOVA test regarding measured values and predicted IPW & IMW according to Pont’s index for male & female Egyptian sample

Parameter	Male(*N* = 82)		Female(*N* = 102)		Test of significance	
	M	SD	M	SD	t-value	Sig.
**SI**	30.86	2.15	30.83	1.96	0.009	0.921
**Measured IPW**	37.55	3.29	37.79	3.01	− 0.520	0.604
**Measured IMW**	47.27	3.31	47.06	2.91	0.470	0.639
**Predicted IPW**	38.57	2.68	38.53	2.45	0.099	0.921
**Predicted IMW**	48.22	3.35	48.17	3.06	0.109	0.913

Notes: Data is mean (M) and standard deviation (SD). The test of significance is one-way ANOVA. SI = sum of incisors. IPW = inter-premolar width. IMW = inter-molar width

**Table 3:** Mean & SD and *P*-value of one-way ANOVA test regarding measured values and predicted IPW & IMW according to Pont’s index for NO and different classes of dental MO in Egyptian individuals

Parameter	Class I *n* = 46		Class II *n* = 46		Class III *n* = 46		Normal occlusion *n* = 46		Total *n* = 184			F	*p*-value
	M	SD	M	SD	M	SD	M	SD		M	SD		
SI	30.3	2.1	30.8	1.8	30.9	2.4	31.5	1.6		30.84	2.04	2.441	0.066
Measured IPW	38.2	2.8	35.7	2.4	38.3	3.9	38.9	2		37.69	3.13	10.879	**< 0.001**
Measured IMW	47.2	3.1	46	2	48.2	3.9	47.2	2.7		47.15	3.08	4.375	**0.005**
Predicted IPW	37.9	2.6	38.5	2.2	38.6	3	39.4	2		38.55	2.55	2.441	0.066
Predicted IMW	47.4	3.2	48.2	2.8	48.2	3.8	49.3	2.5		48.19	3.18	2.447	0.065
(Predicted- Measured) IPW	0.24	3.25	2.83	2.59	− 0.14	4.23	0.56	2.43		0.24	3.25	5.93	**< 0.001**
(Predicted- Measured) IMW	0.95	3.87	1.96	2.99	− 0.34	4.81	2.13	3.71		0.95	3.87	3.061	0.017

Notes: Data is mean (M) and standard deviation (SD). The test of significance is one-way ANOVA. SI = sum of incisors. IPW = inter-premolar width. IMW = inter-molar width

**Table 4:** Post-hoc tests for significant results of one-way ANOVA

Pair	Class I	Class II	Class III	Normal occlusion
**Measured IPW**	38.2 ± 2.8^a^	35.7 ± 2.4^abc^	38.3 ± 3.9^b^	38.9 ± 2^c^
**Measured IMW**	47.2 ± 3.1	46 ± 2^a^	48.2 ± 3.9^a^	47.2 ± 2.7

Notes: IPW = inter-premolar width. IMW = inter-molar width. The test of significance is Games-Howell. Same superscript letters indicate significant difference and different superscript letters indicate insignificant difference

**Fig. 3:**
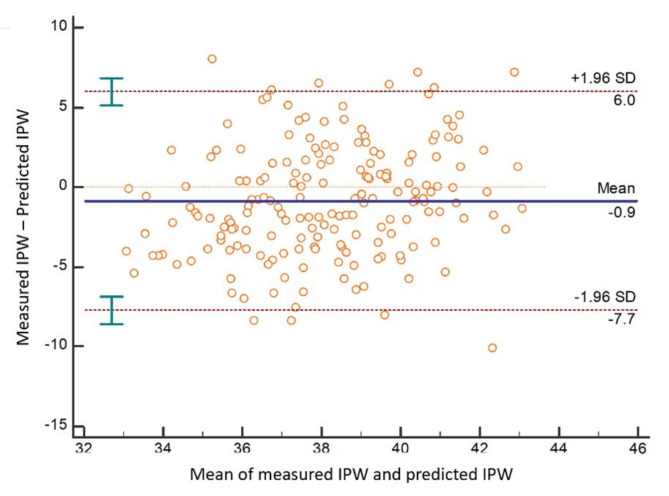
Bland-Altman plot for measured and predicted inter-premolar width (IPW). In this figure, the null hypothesis of no difference (H_0_: Mean = 0) was rejected (*P* = 0.0009)

**Fig. 4:**
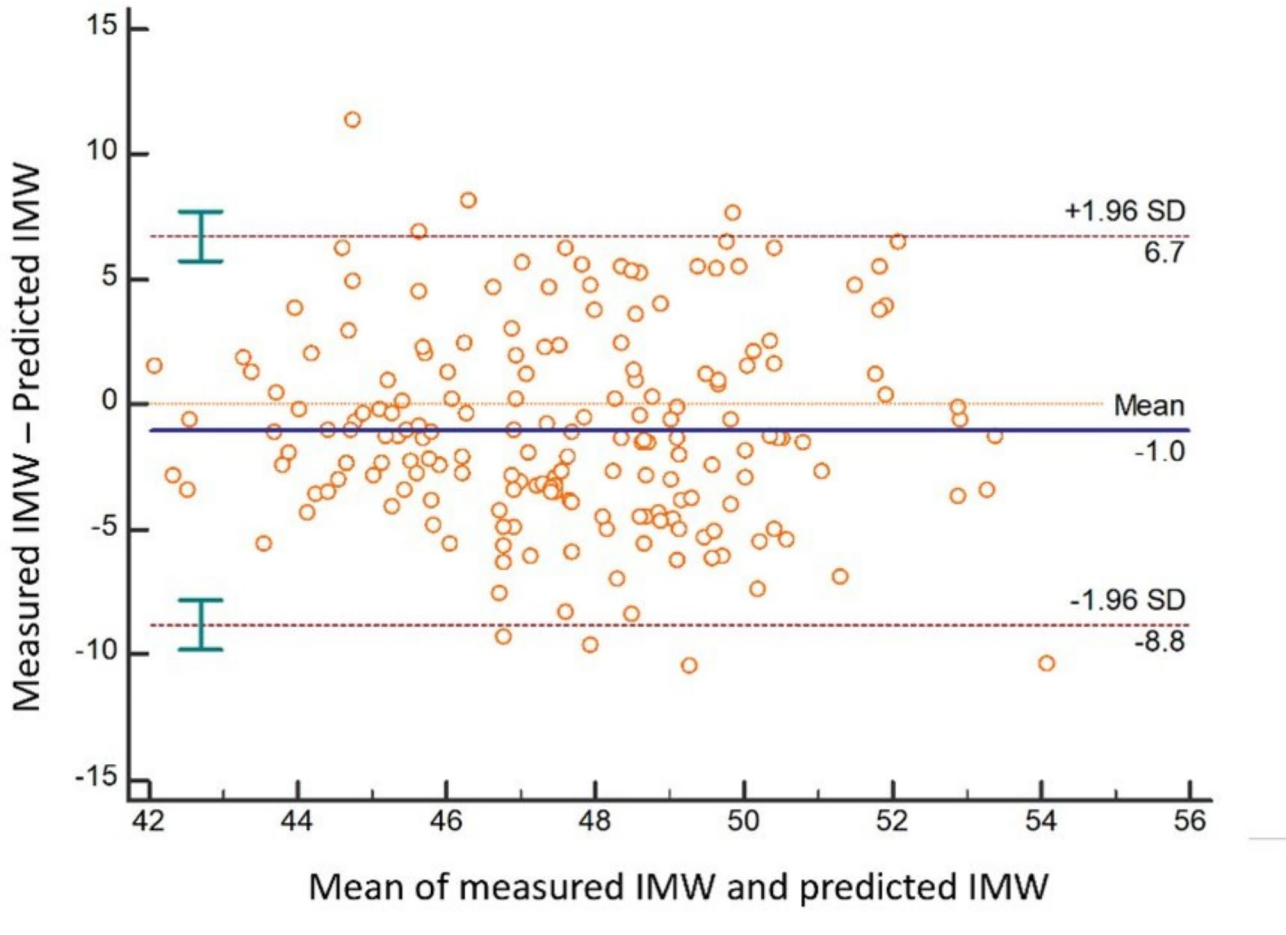
Bland-Altman plot for measured and predicted inter-molar width (IMW). In this figure, the null hypothesis of no difference (H_0_: Mean = 0) was rejected (*P* = 0.0005)

ICC (absolute agreement) between measured and predicted IPW & IMW was determined for NO, Class I, Class II and Class III MO groups (Table 5). The results show that there was a poor absolute agreement between measured and predicted IPW & IMW in NO, Class I, Class II and Class III MO groups (ICC < 0.5). In the upper arch Bland-Altman plots revealed a significant difference between measured and predicted IPW & IMW (*P* = 0.0009 for IPW & *P* = 0.0005 for IMW) (Figs. 3 and 4) confirming the rejection of the null hypothesis of no difference between measured and predicted arch widths based on Pont’s equations and accepting the alternative hypothesis.

**Table 5:** Absolute agreement between measured and predicted IPW & IMW in the four groups

Source	Class I				Class II				Class III				Normal occlusion			
	ICC	95% CI for ICC		*p*-value	ICC	95% CI for ICC		*p*-value	ICC	95% CI for ICC		*p*-value	ICC	95% CI for ICC		*p*-value
		Lower	Upper			Lower	Upper			Lower	Upper			lower	upper	
IPW	0.188	-0.096	0.442	0.960	0.142	-0.076	0.371	**0.043**	0.321	0.047	0.549	**0.012**	0.257	-0.069	0.537	0.061
IMW	0.160	-0.126	0.420	0.135	0.196	-0.050	0.432	**0.029**	0.308	0.031	0.540	**0.015**	− 0.008	-0.241	0.266	0.523

Notes: ICC = intraclass correlation coefficient. CI = confidence interval. IPW = inter-premolar width. IMW = inter-molar width

Table 6 shows Correlations between SI and measured IPW & IMW. A statistically significant positive correlation was detected between SI and measured IPW & IMW of low strength (*r* = 0.256 and 0.206, respectively) in the Egyptian individuals.

**Table 6:** Correlation between sum of MD width of upper incisors and measured IPW & IMW in the Egyptian individuals

Parameter	Total	
	*r*	Sig.
**Measured IPW**	0.256	**< 0.001**
**Measured IMW**	0.206	**0.005**

Notes: r = Pearson’s correlation coefficient. Sig.= *p*-value. The strength of association is low, medium, and large when *r* = 0.1–0.3, 0.3–0.5, and > 0.5, respectively. IPW = inter-premolar width. IMW = inter-molar width

## Discussion

One of the main goals of orthodontics has always been to create an arch form that is stable with proper function and aesthetically pleasing [20]. Various techniques for correcting TDM were described such as lateral enlargement at the mid-palatal suture. The mid-palatal suture starts to fuse as adolescence progresses into adulthood, necessitating the use of stronger orthodontic forces to drive the maxilla into expansion. Therefore, in order to maximize treatment efficacy, it is necessary to precisely diagnose the requirement for transverse maxillary expansion as soon as possible [21]. One of the simple diagnostic tools for assessment of TDM is Pont’s Index [22] Numerous researchers [23–27] have assessed Pont’s index’s clinical applicability on a range of racial and ethnic groups to see if the index might be used on various populations.

This study was designed to ascertain whether Pont’s index is a reliable method for predicting IPW & IMW based on the combined MD dimensions of the maxillary incisors in the Egyptian population. According to previous studies, [28, 29] the accuracy of desktop scanning of plaster models versus direct intraoral scanning did not differ significantly. In comparison to the indirect scanning method, intraoral scanning was found to be less valid and reliable according to Kirschneck et al. [28] Additionally, Murugesan et al. [29] performed a study and compared the values acquired from direct intraoral measurements with the measured values derived from the plaster model, digital models produced by scanning the plaster models, and direct intraoral scanning. There was no statistically significant difference in the measurements between the four approaches. Consequently, using desktop scanner is considered a clinically reliable approach. The outcomes of the study demonstrated that there was a poor absolute agreement between measured and calculated IPW & IMW values based on Pont’s equations in NO and the three classes of dental MO groups. This study revealed that Pont’s index overestimates IPW & IMW for the Egyptian population. This disagreed with Sridharan et al. [16], who finally concluded that Pont’s index may be used with the Tumkur population as they proposed that Pont’s predicted values and mean measured values did not differ significantly. Dhakal et al. [15] concluded that Pont’s equations is applicable to Nepalese individuals as there was a definite correlation between SI and IPW & IMW, in agreement with Pont’s values. On the contrary, this study agreed with Worms et al. [7] who proposed there was a significant difference between measured and calculated IPW and IMW in Navajo sample. The observed measurements were less than the calculated measurements for most participants which suggested that the Navajo individuals had narrower arches than those calculated based on Pont’s equations. This also agreed with the outcomes of Nimkarn et al. [10] and Celebi et al. [11] who discovered that the IPW and IMW needed to relief dental crowding in Caucasian and Turkish individuals, respectively are overestimated by Pont’s equations. Dalidjan et al. [30] and Lew [31] showed underestimation in values obtained using Pont’s index while Kim et al. [26] found both over and underestimation. Low correlations were discovered by Rathi et al. [18] between the observed and Pont’s predicted IPW (*r* = 0.364) and IMW (*r* = 0.238) who proposed that Pont’s analysis has no chance to be a clinically valuable indicator for arch widths in Pakistani population.

No significant difference was detected between genders for SI and measured IPW & IMW in the current study similar to the results of a study conducted by Alam et al. [17] on Bangladeshis. This matches up with the findings of Bishara et al. [32] who examined the tooth sizes of three groups from Mexico, Egypt, and the United States and discovered no gender-specific variations in maxillary incisor widths. There were no significant variations in maxillary incisor widths between the sexes as reported by Celebi et al. [11] and Al-Omari et al. [9] who conducted studies on Turkish and Jordanian populations, respectively. This also agreed with the outcomes of Al-Khateeb and Abu-Alhaija, [33] who discovered no significant differences in maxillary incisors MD widths between Jordanian males and females with a Class I occlusion. This contradicts the results of Hattab et al. [34] and Dungarwal et al. [20] who concluded that males have considerably wider maxillary incisor widths than females, according to statistical analysis. The results of a study conducted by Dungarwal et al. [20] on Martha population showed that IPW and IMW were larger in males than in females which is in agreement with the outcome of a study on a British sample [35] and the Saudi population [36] but this disagreed with the results of the current study and a previous study conducted on the Egyptian population [37] which revealed no significant difference between males and females in IPW and IMW.

The current study demonstrated a significant correlation of low strength between SI and measured IPW (*r* = 0.256) & measured IMW (*r* = 0.206) in overall Egyptian sample which agreed with the results of a study conducted on the Kurdish population by Aziz et al. [38] and revealed that Pearson correlations between IMW, IPW and SI were found to be equal to 0.33 and 0.32, respectively, which reflected weak positive significant correlations. In the current study on the Egyptian population, a poor agreement was found between the actual measurements and the values calculated by Pont’s equations which demonstrated that Pont’s index wasn’t a reliable way to estimate arch width values for orthodontic treatment. Consequently, new formulas were created that used coefficients of regression (r) to predict IPW & IMW by knowing the dependent variable, SI. Using linear regression test, the following equations were created for the Egyptian population:

1. *Predicted IPW* = 27.9 + (0.33 × SI).
2. *Predicted IMW* = 44.28 + (0.08 × SI).

## Conclusion

For determining the appropriate dental arch width in the Egyptian population, Pont’s index is not a valid method as there was poor absolute agreement between measured and calculated IPW & IMW based on Pont’s equations in NO, class I, class II and class III MO groups, so corrected Pont’s equations should be used to predict IPW & IMW to aid in the diagnosis and treatment planning of TDM in the Egyptian population.

### Recommendation

Application of the corrected Pont’s equations on a larger scale of the Egyptian population.

## Data Availability

All the datasets used and analyzed during the current study are available from the corresponding author on reasonable request.

## Abbreviations

IMW: Inter-molar width
IPW: Inter-premolar width
MD: Mesio-distal
MO: Malocclusion
NO: Normal occlusion
SI: Sum of incisors
TDM: Transverse deficiency of the maxilla
